# Behavior Change Techniques in Physical Activity Interventions Targeting Overweight and Obese Children and Adolescents: A Systematic Review

**DOI:** 10.3390/bs14121143

**Published:** 2024-11-28

**Authors:** Sanying Peng, Ahmad Zamri Khairani, Fang Yuan, Abubakar Rabiu Uba, Xiaoming Yang

**Affiliations:** 1 Department of Physical Education, Hohai University, Nanjing 210024, China; 2 School of Educational Studies, University Sains Malaysia, Penang 11800, Malaysia; ahmadzamri@usm.my; 3 College of International Languages and Cultures, Hohai University, Nanjing 210024, China; yuanf@hhu.edu.cn; 4 Department of Education, Sule Lamido University, Kafin Hausa 731102, Nigeria; abubakar.rabiu@slu.edu.ng; 5 Physical Education College, Shanghai University, Shanghai 200444, China; 770721@shu.edu.cn

**Keywords:** behavior change technique, obese, overweight, physical activity, systematic review

## Abstract

Substantial evidence globally confirms the benefits of physical activity (PA) interventions for the physical and mental health of overweight and obese children and adolescents. However, current research has yet to determine which behavior change techniques (BCTs) are most effective in PA interventions for this population. This systematic review aims to evaluate the application of BCTs in PA interventions for overweight and obese children and adolescents and to identify the most effective BCTs using the promise ratio. Five electronic databases (PubMed, Embase, Cochrane Central, Web of Science, and PsycINFO) were searched up to 31 May 2024, to identify intervention studies meeting the eligibility criteria for promoting PA in the target population. Thirteen studies were included (nine randomized controlled trials and four quasi-experimental studies). The quality of the included studies was assessed using a revised version of the Cochrane Risk of Bias tool and the Risk of Bias in Non-randomized Studies tool. Among the 24 BCTs implemented, the most frequently applied were goal setting (behavior), instruction on how to perform the behavior, feedback on behavior, and self-monitoring of behavior. Action planning, social support, and material incentives showed the most significant potential to promote PA. These findings provide valuable insights for designing future PA interventions for this group, with the potential to improve health outcomes and enhance PA participation among obese children and adolescents.

## 1. Introduction

The global prevalence of overweight and obesity among children and adolescents has reached concerning levels. According to the World Health Organization (WHO), the number of overweight and obese children and adolescents aged 5 to 19 exceeded 390 million by 2022, a substantial increase from 32 million in 1990 [1]. This sharp rise is associated with significant health risks, including type 2 diabetes, cardiovascular diseases, hypertension, and metabolic syndrome, conditions now emerging even in younger populations [2,3]. Moreover, these health problems often persist into adulthood, exacerbating the long-term public health burden [1].

In response to this growing health crisis, increasing physical activity (PA) levels has been identified as a primary intervention strategy to alleviate both the physical and psychological impacts of childhood obesity. PA plays a well-documented role in weight control and improves physical health markers, including insulin sensitivity, cardiovascular function, and muscle strength [4,5]. Beyond physical benefits, PA is crucial for enhancing psychological well-being, which is often compromised in overweight and obese youth. Regular PA engagement is associated with reduced anxiety, depression, and psychological distress, along with improved self-esteem, social interaction, and cognitive function [6,7,8]. For children and adolescents facing stigma or bullying due to their weight, PA also fosters resilience and enhances quality of life [9].

Despite the well-established physical and psychological benefits of PA, a considerable number of overweight and obese children and adolescents fail to meet the WHO recommendation of at least an average of 60 min of moderate-to-vigorous PA per day [10]. Research highlights consistently low adherence to PA guidelines in this population, with contributing factors including a lack of motivation, environmental barriers, and social challenges [11]. This discrepancy between the recognized benefits of PA and actual participation rates highlights the urgent need for targeted interventions that are both effective in increasing PA levels and sustainable over the long term.

To address this issue, researchers have developed various interventions aimed at promoting PA among these populations. While some interventions have successfully increased PA [12,13], others have shown limited or no effects [14,15]. However, these studies generally lack specificity regarding the embedded behavior change techniques (BCTs), defined as distinct strategies intended to influence specific behavioral determinants, such as motivation or self-efficacy, to support sustained behavior change [16]. This lack of specificity obscures which elements may most effectively drive behavior change within PA components.

Previous reviews offer mixed findings on the effectiveness of PA and lifestyle interventions for this demographic. For instance, Jurado-Castro et al. and Podnar et al. reported favorable outcomes in school-based interventions for BMI and PA [17,18], yet neither study examined the role of specific BCTs, limiting insights into the psychological mechanisms underlying effective interventions. Salam et al. highlighted the benefits of multi-component interventions that combined diet, exercise, and behavioral therapy, though without isolating the contributions of individual BCTs [19].

Furthermore, Nooijen et al. observed that standard PA interventions often fail to significantly improve objectively measured PA outcomes in overweight and obese children [20], in contrast to positive effects seen in non-obese counterparts, as reported by Metcalf et al. [21]. This discrepancy suggests unique psychological and behavioral barriers among overweight and obese youth that may limit the effectiveness of traditional PA interventions. These findings underscore a gap in the literature regarding specific BCTs that could enhance PA engagement, highlighting the need for research investigating BCTs’ roles in this context.

The BCTs taxonomy, developed by Michie and colleagues [16], has played a pivotal role in identifying the active components of behavioral interventions across various health domains, including PA [22]. This taxonomy provides a systematic framework for isolating the key elements that drive behavior change. BCTs have been extensively applied across different populations, including adults [23], cancer survivors [24], individuals with chronic conditions [25], and older adults [26], consistently demonstrating their effectiveness in identifying and refining key elements that drive behavior change in PA interventions. Techniques such as goal setting, self-monitoring, and action planning are particularly effective in supporting sustained improvements in PA, with solid evidence backing their use across diverse groups [26,27,28]. However, to date, no systematic review has specifically applied the BCTs framework to PA interventions targeting overweight and obese children and adolescents. Considering this population’s unique motivational and psychological barriers, adapting BCTs may be critical for achieving lasting impact.

Therefore, the aim of this review is twofold: first, to identify and evaluate the application of BCTs in PA interventions targeting overweight and obese children and adolescents, and second, to determine the most promising BCTs in PA interventions by utilizing the promise ratio as an evaluation metric. Through a systematic review, this study provides valuable insights into which BCTs hold the most significant potential for increasing PA levels within this population, guiding the development of future interventions.

## 2. Methods

This systematic review was conducted following the PRISMA guidelines [29] and the Cochrane Handbook [30]. The protocol was registered with the International Prospective Register of Systematic Reviews (PROSPERO) under registration number CRD42024576298.

### 2.1. Search Strategy

The search strategy was developed based on the research topic and retrieval methods from relevant reviews (as shown in S1 of the Supplementary Materials). A comprehensive search was conducted across five electronic databases: PubMed, Embase, Cochrane Central, Web of Science, and PsycINFO, using predefined search strategies. The search covered the period from the inception of each database until 31 May 2024. No language restrictions were applied during the search.

The search scope included “titles, abstracts, and keywords” or equivalent terms. The search terms were organized into three key domains: (1) “children”, “adolescents”, “obesity”, and “overweight”; (2) “physical activity” and “exercise”; and (3) “intervention”, “trial”, and “experiment”. Boolean operators were effectively applied to combine and structure the search terms. A recursive search of references from related reviews was also performed to ensure the inclusion of all relevant studies.

### 2.2. Eligibility Criteria

The inclusion criteria for the studies were established according to the PICOS framework (Participants, Interventions, Comparators, Outcomes, and Study design):

Participants: Children and adolescents aged 6–18 years who are classified as overweight or obese (Body Mass Index [BMI] above the 85th percentile). Participants must be capable of engaging in PA interventions. Those with physical disabilities or severe mental illnesses were excluded.

Interventions: Studies primarily focused on promoting PA were included. The interventions required clear descriptions and explanations, and PA outcomes needed to be reported before and after the intervention. Studies centered primarily on weight loss, weight control, or dietary regulation were excluded.

Comparators: Any control group was considered, including but not limited to usual care and waitlist controls.

Outcomes: Any PA outcomes measured by subjective questionnaires or objective measurement tools were included.

Study design: Any intervention study aimed at promoting PA was included, such as randomized controlled trials (RCTs), pilot RCTs, cluster RCTs, quasi-experimental studies, and feasibility trials.

### 2.3. Study Selection

All records were entered into EndNote 20 software (Thomson ISI Research Soft, Philadelphia, PA, USA). After removing duplicates and irrelevant records, two authors independently screened the remaining records in three stages. The first stage involved screening titles and abstracts. In the second stage, a backward citation search of references from relevant studies was performed, and any studies meeting the inclusion criteria were added. In the third stage, full-text screening and evaluation of the selected studies from the previous stages were conducted. To minimize bias, any disagreements at each stage were resolved through group discussions, with a third author acting as a judge when necessary.

### 2.4. Data Extraction

Two authors independently extracted data from the included studies using a data extraction form designed by the first author (PSY). To ensure data extraction accuracy, three studies were randomly selected to compare consistency. Once a 90% agreement rate was achieved, formal data extraction commenced. PSY verified the extracted data for accuracy and completeness, and a third author resolved any discrepancies. The extracted information included essential study characteristics (sample size, age, BMI, country, study design), intervention characteristics (content, delivery mode, intervention duration, and theoretical framework), and outcome details (PA measurement tools and outcomes). Additionally, the effects of the interventions were summarized. 

### 2.5. Risk of Bias Assessment

The revised Cochrane Risk of Bias 2 (ROB 2) tool [31] was used to assess bias in RCTs, while the Risk of Bias in Non-randomized Studies (ROBINS-I) tool [32] evaluated bias in non-randomized studies. The ROB 2 evaluates the risk of bias across five domains: (1) bias arising from the randomization process, (2) bias due to deviations from the intended interventions, (3) bias due to missing outcome data, (4) bias in the measurement of the outcome, and (5) bias in the selection of the reported result. Each domain is rated as “low risk”, “some concerns”, or “high risk”. Similarly, the ROBINS-I tool assesses the risk of bias in non-randomized studies across seven domains: (1) bias due to confounding, (2) bias in the selection of participants into the study, (3) bias in the classification of interventions, (4) bias due to deviations from intended interventions, (5) bias due to missing data, (6) bias in the measurement of outcomes, and (7) bias in the selection of the reported result.

Regarding the overall risk of bias in the included studies, a study was categorized as having a low risk of bias if all domains were assessed as low risk. In contrast, a high risk of bias was assigned if any domain was judged to have a high risk. Studies were considered to have a moderate risk of bias if at least one domain was identified as having “some concerns”, provided no domain was rated as high risk.

### 2.6. BCTs Coding

Based on Michie et al.’s BCTs taxonomy V1 [16], two authors (PSY and YF) independently extracted and coded the BCT elements from the PA interventions in the included studies (including any BCTs applied to the control groups). To ensure accuracy and completeness in data extraction, both coders underwent training on the official BCT website (https://www.bct-taxonomy.com/ (accessed on 12 November 2024)) before initiating the extraction process. A random selection of three studies was used for a pre-extraction trial, and formal extraction commenced only after achieving an agreement rate of 95% or higher. Any discrepancies encountered during the coding process were resolved through group discussions, with a third author acting as a judge when necessary.

### 2.7. BCTs Synthesis and Analysis

To explore the potential contribution of specific BCTs to PA promotion, we analyzed the relationship between different BCT types and the effects of PA intervention. This approach was developed by Gardner and colleagues [33] and is called “promise”, and it has been widely adopted in BCT-related PA intervention studies [25,28,34]. First, each study was categorized into one of three levels of promise based on the intervention’s effect: “no promising”, “quite promising”, or “very promising”. “No promising” refers to interventions where the PA levels of the intervention group did not show statistically significant improvements compared to baseline, nor significant differences from the control group at post-intervention. “Quite promising” indicates that the intervention group showed substantial within-group PA improvement or that their post-intervention PA levels were higher than those of the control group. “Very promising” refers to interventions where the intervention group demonstrated significant post-intervention PA improvements compared to the control group.

Furthermore, the promise ratio was calculated to assess the contribution of individual BCTs to the interventions. This ratio is the sum of studies classified as “quite promising” or “very promising” for a specific BCT, divided by the number of “no promising” studies that applied the same BCT. If no “no promising” studies were present, the total number of promising studies (both “quite promising” and “very promising”) was used to represent the promise ratio. Following the evaluation criteria of previous studies, a BCT was considered promising if its promise ratio was greater than or equal to 2.

## 3. Results

### 3.1. Searching Results

**Figure 1:**
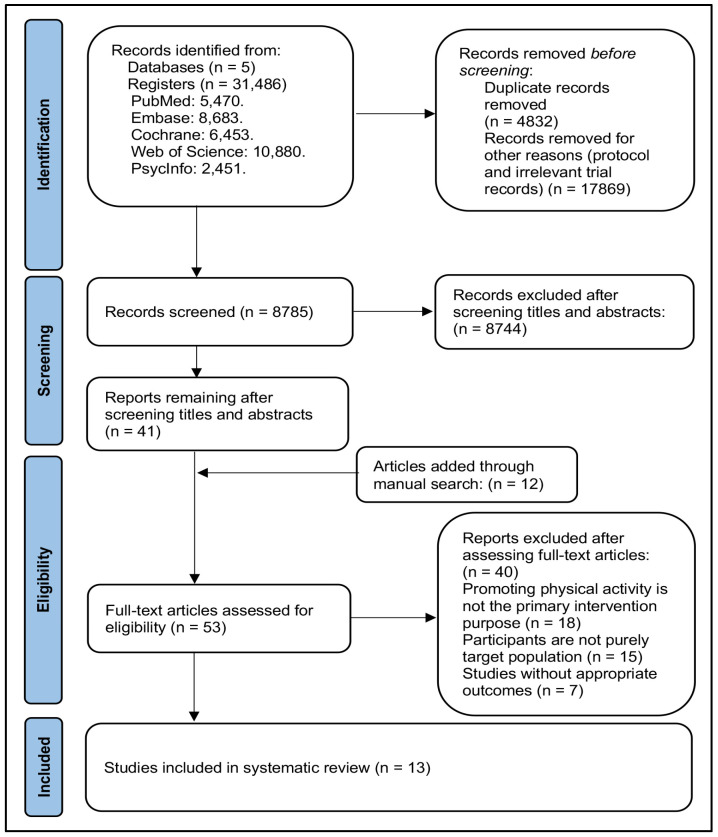
PRISMA flowchart for study selection.

The search across five databases yielded a total of 31,486 records. After removing 4832 duplicates and excluding 17,869 protocols and irrelevant records without specific experimental details, 8785 records remained. Following further screening of titles and abstracts, 41 records were selected for full-text review. Additionally, twelve studies were identified through manual searches of related reviews’ reference lists and were subjected to full-text screening. After thoroughly reviewing the 53 full-text articles, thirteen studies [12,13,14,15,35,36,37,38,39,40,41,42,43] met the inclusion criteria and were included in the systematic review. The detailed screening process is shown in Figure 1.

### 3.2. Characteristics of Included Studies

The thirteen included studies involved a total of 773 participants, with sample sizes ranging from 28 to 105. The average age of participants ranged from 9.2 to 16.0 years. All participants were children and adolescents classified as overweight or obese, with BMI values ranging from 23.1 to 36.1 and a minimum BMI percentile of 94. All thirteen studies were conducted in developed countries, with seven in the United States [12,14,15,37,39,40,42], two in Canada [38,43] and Sweden [35,36], respectively, and one each in Italy [13] and Finland [41]. Of the included studies, nine were RCTs [12,14,35,36,38,39,41,42,43], and four were quasi-experimental studies [13,14,15,40]. The essential characteristics of the included studies are summarized in Table 1.

### 3.3. Intervention Description

The thirteen included studies employed various interventions aimed at promoting PA. Among them, six studies demonstrated very promising intervention effects [12,13,38,39,40,42], two studies showed quite promising effects [37,43], and five studies were rated as having no promising effects [14,15,35,36,41]. Detailed intervention characteristics are presented in Table 1.

Regarding intervention delivery, only three studies [12,36,38] used face-to-face sessions as the sole method of delivering the intervention. All other studies employed a combination of delivery modes. Nine studies [13,14,15,35,37,39,40,41,42] incorporated digital health technology as part of the intervention, while one study combined individual sessions with family and group support [43]. The duration of the interventions varied from one month to 24 months.

Seven studies explicitly used theoretical frameworks to guide the intervention design [12,13,15,37,41,42,43], with Social Cognitive Theory (SCT) being the most frequently cited [13,15,43].

Three studies used self-reported PA measures [13,39,43], while the rest employed objective instruments, with accelerometers being the most widely used, appearing in six studies to measure different intensities of PA [12,15,37,38,40,42].

### 3.4. Risk of Bias

Using the ROB-2 tool to assess the ROB for eight RCTs, two studies were rated as having low risk [12,35], three as high risk [14,36,42], and three as moderate risk [38,39,41]. In the five evaluation domains, the randomization process was unclear in two studies [36,39], while the other six studies were rated as low risk. In the domain of missing outcome data, three studies were judged to have a high risk of bias [14,36,42]. One study was considered high risk for the five non-randomized controlled trials evaluated using the ROBINS-I tool [43], as it was rated high risk in three domains: confounding, missing data, and selection of the reported result. The overall ROB for the other studies was rated as moderate risk. A summary of the quality assessment of the thirteen studies is presented in Table 1, while detailed ROB evaluations are available in S2 of the Supplementary Materials.

### 3.5. Behavior Change Techniques

Among the thirteen interventions, 24 out of the 96 BCTs were used. The number of BCTs applied in each study ranged from 5 to 11, averaging 7.5. The extracted BCT information from each study is presented in Table 1. The coding details of the BCTs extracted from each study are provided in S3 of the Supplementary Materials.

**Figure 2:**
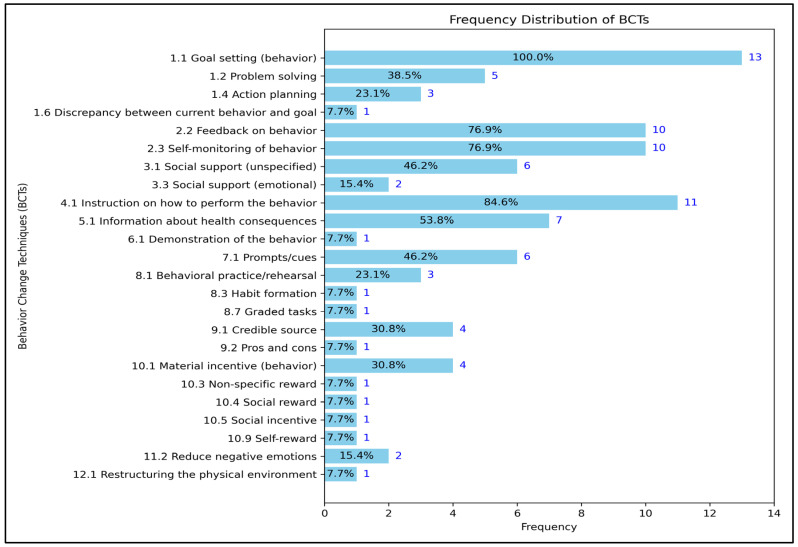
The frequency of BCTs used in the included studies.

The most commonly used BCTs across all studies were goal setting (behavior), which was applied in every study, and instruction on how to perform the behavior, which was used in eleven studies. Additionally, feedback on behavior and self-monitoring of behavior were employed in ten studies. At the same time, information about health consequences, prompts/cues, and social support (unspecified) were used in nearly half of the studies. BCTs such as the discrepancy between current behavior and goal, demonstration of the behavior, habit formation, graded tasks, pros and cons, non-specific reward, social reward, social incentive, self-reward, and restructuring the physical environment were only used in a single study each. The frequency of BCT usage in the included studies is detailed in Figure 2.

Table 2 illustrates the relationship between BCTs, intervention effectiveness, and the calculated promise ratios. The BCTs with a promise ratio greater than 2, indicating a significant recent intervention effect, were action planning, social support (unspecified), and material incentive (behavior). Demonstration of the behavior, habit formation, graded tasks, pros and cons, social reward, self-reward, and restructuring the physical environment were each found only once in very promising studies. The discrepancy between current behavior and goal, non-specific reward, and social incentive appeared only once in quite promising studies. There is no BCT only in non-potential studies. Additionally, BCTs with a promise ratio greater than 1 included goal setting (behavior), problem solving, feedback on behavior, self-monitoring of behavior, instruction on how to perform the behavior, and information about health consequences.

## 4. Discussion

This systematic review identified the application of 24 different BCTs in interventions aimed at promoting PA among overweight and obese children and adolescents. The most frequently employed BCTs were goal setting (behavior), instruction on how to perform the behavior, feedback on behavior, self-monitoring of behavior, information about health consequences, prompts/cues, and social support (unspecified). These findings emphasize the importance of understanding which BCTs are most effective in promoting PA among this group. Action planning, social support (unspecified), and material incentives (behavior) emerged as particularly promising for increasing PA, highlighting their value in health behavior interventions.

The identification of 24 BCTs in this review is consistent with findings from similar age groups, where Seims et al. extracted 26 BCTs in family-based PA interventions [44] and Al-walah et al. reported 27 BCTs for preschool children’s PA interventions [45]. Moreover, Sivaramakrishnan et al. uncovered 35 BCTs in interventions for children with chronic conditions [25], while Watson et al. documented up to 46 BCTs in interventions for those with spinal cord injuries [46]. O’Dwyer et al. also observed 15 of the 16 BCT categories in the BCTs taxonomy V1 during PA interventions for adults with fibromyalgia [47]. These variations in BCT counts suggest that specialized patient groups may benefit from a greater variety of techniques. Still, the mere number of BCTs included in an intervention does not guarantee effectiveness. However, previous studies have shown no clear association between the number of BCTs included in an intervention and its effectiveness [48,49]. Identifying the specific types of BCTs, their frequency of use, and their relationship to the effectiveness of the intervention may offer a more promising approach to understanding the role of BCTs in PA interventions [50,51].

Goal setting (behavior) was the most utilized BCT in PA interventions for our age-similar cohort. It also featured prominently in varied demographic studies, including those involving breast cancer survivors [28], adults with asthma [52], older adults [27], and individuals with spinal cord injuries [46]. The frequent use of goal setting in PA interventions can be attributed to its ability to provide structure, clear targets, and intrinsic motivation, making it effective across different groups [48,53]. Moreover, over half of the reviewed studies implemented instructions on how to perform the behavior, feedback on behavior, and self-monitoring of behavior, which also frequently appear in PA enhancement studies involving similar age groups [25,44,45], enhancing autonomy and self-regulation. These techniques, by providing step-by-step guidance and immediate feedback, contribute to building a sense of control and improving adherence to PA. Interestingly, information about health consequences, frequently applied in this study, was absent in similar demographic research. A possible explanation is that, for children and adolescents who are obese or overweight, increasing their understanding of the health benefits of PA—such as losing weight, improving physical fitness, reducing the risk of chronic diseases, and enhancing the quality of life—can significantly boost their motivation to participate in PA [54,55]. Furthermore, individuals in this age group tend to be more responsive to health-related information, so providing precise details on the consequences of health behaviors can effectively encourage them to adopt healthier lifestyle changes.

Action planning, social support (unspecified), and material incentive (behavior) were assessed as promising BCTs for enhancing PA interventions, as determined using the promise ratio. Action planning enhances the implementation of PA by encouraging individuals to develop specific action steps. This approach helps individuals determine when, where, and how to engage in activities, thus increasing the predictability and feasibility of actions and effectively bridging the gap between intention and behavior [56,57]. This BCT was also identified as promising for interventions in the studies conducted by Al-walah et al. [45], Sivaramakrishna et al. [25], and Tyson et al. [52], all of which utilized the promise ratio method. Social support (unspecified) enhances individuals’ motivation and ability to engage in PA by offering emotional or practical assistance from friends, family, or community members [16]. This support helps individuals overcome barriers to PA by fostering a sense of involvement and belonging and providing necessary resources or encouragement [58]. Its broad and flexible nature allows it to meet the diverse needs of different individuals, particularly adolescents and children. This BCT was also identified as promising for interventions in studies by Sivaramakrishnan et al. [25] and Carlin et al. [59]. While other similar studies have not found evidence supporting material incentive (behavior) as a promising approach in PA interventions, the study by Whatnall et al. explicitly states that in PA interventions among youth, the BCT of material incentive (behavior) is most strongly associated with the most significant effect enhancement [60]. The significance of using material incentives to promote PA in obese youth is reflected in their ability to satisfy youth’s immediate gratification and anticipated rewards directly [61]. Obese children typically exhibit a lack of established positive behavioral habits and sustained motivation. Material rewards, such as small gifts or additional game time, can immediately reinforce positive behaviors and motivate participation in PA. By offering a sense of achievement and control, this approach helps children develop positive behavioral patterns tailored to their specific cognitive and behavioral needs, promoting lasting changes in healthy behaviors [62].

Six of the included studies identified PA interventions as promising. In these studies, various BCTs were delivered through face-to-face sessions or hybrid approaches, with none employing a purely remote intervention model. For example, Henderson et al. [38] provided one-on-one exercise counseling to educate obese participants, fostering motivation and tailoring goals and strategies based on each participant’s stage of behavior change. Similarly, Suksong et al. conducted a PA intervention that enhanced participants’ self-efficacy by gradually increasing activity difficulty and combining in-person with remote support, ultimately helping participants achieve their PA goals [42]. These studies underscore the critical role of face-to-face interaction in delivering interventions, noting that in-person delivery remains significantly advantageous despite the advancements in digital health and remote technologies.

Digital health technologies have been recognized for their role in promoting PA. However, in this study, digital methods were less effective than face-to-face approaches. This may be due to insufficient integration of BCTs into digital tools, often reducing them to feedback mechanisms rather than comprehensive intervention components. Despite these shortcomings, recent studies have shown promising outcomes for digital approaches like exergames and active video games, which effectively promote PA among overweight and obese youth [63,64]. These games align with this group’s high screen time and sedentary habits, making them particularly suitable for intervention [63]. The embedded BCTs, such as self-reward, self-monitoring of behavior, and action planning, enhance participant engagement and adherence to PA programs. The interactive, rewarding nature of these games may better address the needs of this population and foster more sustainable PA behaviors. Future research should further explore how BCTs can be effectively embedded into eHealth interventions, focusing on optimizing these elements across various environments to support long-term behavior change.

On the other hand, five studies were classified as non-promising. These interventions used hybrid delivery methods; no clear association was found between effectiveness and duration. Although the interventions in the two studies by Backlumd and colleagues [35,36] and the study by Rubin et al. [15] are characterized by their innovative and appealing content, they lack sustained reinforcement of the interventions. The studies by Currie et al. [14] and Ruotsalainen et al. [41] attempted to incorporate family and community support within the interventions. Still, this support often failed to be fully mobilized, which may have limited the impact on behavior change. Additionally, the low frequency of monitoring and feedback mechanisms and the mismatch between BCTs and intervention objectives could also constrain the effectiveness of the interventions.

From the theoretical foundation of the interventions, studies supported by theory exhibit a higher efficacy rate (6/7) compared to those without theoretical support (2/6), indicating more effective outcomes that are supported by Gourlan et al. [65]. However, given this research’s limited number of studies, it is insufficient to determine conclusively whether theory-supported interventions are more effective. The critical determinants of PA interventions still require an emphasis on the effective alignment of theory-driven BCTs with the objectives of the intervention and their orderly execution within appropriate contexts [28,45].

The findings of this review have significant practical implications for public health and education, particularly for interventions targeting overweight and obese youth. BCTs such as goal setting, action planning, and social support are crucial in designing effective interventions that provide structured and achievable strategies to enhance engagement in PA [50]. Incorporating these BCTs into public health projects and educational policies, especially within school-based programs, can establish systematic frameworks that promote sustained PA participation. This approach not only helps reduce the prevalence of obesity among youth but also integrates PA into educational settings, promoting overall well-being. Future research should further explore the characteristics and effectiveness of BCTs across different settings, such as schools, families, and community environments, to better tailor interventions to specific contexts.

This review systematically assessed BCTs employed in PA interventions for overweight and obese children and adolescents. Despite a rigorous methodological approach, several limitations must be acknowledged regarding the scope and methodology of this review. First, the limited number of included studies restricts broader generalizations, particularly since most were conducted in developed countries, which may limit applicability to diverse global populations, especially in low- and middle-income countries. Second, the quality of the studies was moderate, which affected the robustness of the evidence. Third, methodological heterogeneity, such as using self-reported and objective PA measures, made comparisons difficult and prevented meta-analysis, which hindered precise quantification of the BCT-PA relationship. Fourth, BCT identification was based on published descriptions, which introduces potential bias due to inconsistent reporting practices. Finally, using the promise ratio offered valuable insights; still, it focused only on immediate impacts without adequately capturing sustainability or long-term behavior change. It did not account for potential declines in PA that could obscure differences between intervention groups. Overall, while this review provides an initial framework for understanding the effectiveness of specific BCTs, the findings should be interpreted cautiously due to the limitations in study scope, methodological diversity, and analytical approaches.

## 5. Conclusions

This study is the first systematic review to examine the application of BCTs in PA interventions targeting obese or overweight children and adolescents. The findings highlight similarities and differences compared to previous research regarding the types of BCTs used and their contribution to PA interventions. Effective strategies for designing interventions for obese children include setting specific exercise goals, developing detailed plans to promote self-regulation, and enhancing self-efficacy through comprehensive emotional and material support. Additionally, offering tangible rewards can motivate participation and adherence to PA. In the context of rising sedentary behavior and the increasing global prevalence of childhood obesity, these findings provide targeted guidance for developing PA interventions for obese children and adolescents. They also offer strong evidence to inform public health practices and policy-making. The clinical relevance of these findings lies in their potential to reduce sedentary behaviors and promote healthier lifestyles, which are critical in managing and preventing obesity among children and adolescents. Future research should explicitly focus on integrating these findings into clinical and community settings, emphasizing public health impacts. Given the limitations of this study, future research on PA promotion for this population should adopt a broader scope. Intervention strategies should be more precisely aligned with specific objectives, and more detailed reporting on the dosage, frequency, and implementation of interventions is needed. Moreover, theoretically supported and digital health intervention approaches are promising for future applications.

## Figures and Tables

**Table 1:** Characteristics of included studies.

Study	Study Characteristic	Intervention	Duration	Theory Used	BCTs	PA Instrument	PA Outcome	Potential to Increase PA	ROB	
									Tool	Appraisal
Backlund et al., 2011a [35]	N = 105; Mean Age: IG = 10.5(1.13), CG = 10.6(1.02); BMI: 23.1(2.65); Country: Swedish; RCT	Content: A 2-year lifestyle intervention Delivery mode: Face-to-face sessions + Internet	24 Months	No	1.1; 2.2; 2.3; 4.1; 8.1; 9.1	SenseWear PRO2 Armband	Steps and METs	Non	The revised ROB 2	L
Backlund et al., 2011b [36]	N = 105; Mean Age: IG = 10.5(1.13), CG = 10.6(1.02); BMI: 23.1(2.65); Country: Swedish; RCT	Content: A 1-year lifestyle intervention Delivery mode: Face-to-face sessions	12 Months	No	1.1; 2.3; 4.1; 5.1; 7.1; 8.1; 9.1	SenseWear Pro2 Armband	Steps and METs	Non	The revised ROB 2	H
Cummings et al., 2022 [37]	N = 28; Mean Age: 14.81; BMI: 97.07th percentile; Country: USA; Quasi-experiment	Content: A digital health program Delivery mode: Combination of digital remote technology (Fitbit, mobile app, SMS) with in-person support and monetary incentives	12 W	SDT	1.1; 1.6; 2.2; 2.3; 4.1; 7.1; 10.1; 10.3; 10.5	Fitbit Charge	Steps and active minutes	Quite	ROBINS-I	M
Currie et al., 2017 [14]	N = 64; Mean Age: 14.4(1.92); BMI: >95th percentile; Country: USA; Pilot RCT	Content: A low-dose PA intervention Delivery mode: Combination of remote communication methods and printed materials	7 W	No	1.1; 1.2; 1.4; 2.2; 3.3; 5.1; 9.1; 11.2	Pedometer	Steps	Non	The revised ROB 2	H
Gourlan et al., 2013 [12]	N = 54; Mean Age: 13(1.66); BMI: 29.57(5.34); Country: USA; RCT	Content: Motivational interviewing as intervention Delivery mode: Face-to-face sessions	6 Months	SDT	1.1; 1.2; 2.2; 2.3; 3.3; 4.1; 5.1; 9.2;	Accelerometer	Total energy expenditure	Very	The revised ROB 2	L
Henderson et al., 2010 [38]	N = 31; Mean Age: IG = 16.0(1.2), CG = 15.3(1.1); BMI: 36.1(6.9); Country: Canada; Pilot RCT	Content: Exercise consultation Delivery mode: Face-to-face sessions	3 Months	TTM	1.1; 1.2; 1.4; 4.1; 5.1; 9.1	Accelerometer	LPA, MPA, and VPA	Very	The revised ROB 2	M
Maloney et al., 2012 [39]	N = 65; Mean Age: IG = 12.9(2.36), CG = 11.73(2.38); BMI: > 96th percentile; Country: USA; RCT	Content: Exergaming with “Dance Dance Revolution” Delivery mode: Combination of technology-based home activities and remote monitoring	12 W	No	2.2; 2.3; 3.1; 7.1; 5.3;12.1	Self-reported frequency of MVPA	Self-reported frequency of MVPA	Very	The revised ROB 2	M
Morano et al., 2020 [13]	N = 64; Mean Age: 11.3(0.5); BMI: >95th percentile; Country: Italy; Quasi-experiment	Content: School-based multi-component intervention Delivery mode: Combination of in-person group sessions, structured physical activities, and self-monitoring	7 Months	SET, SCT	1.1; 1.2; 2.3; 3.1; 4.1; 5.1; 8.3; 10.9	The PA Questionnaire for Older Children (PAQ-C)	Total scores of all activities	Very	ROBINS-I	M
Oreskovic et al., 2016 [40]	N = 60; Age: 10–16; BMI: 94th percentile; Country: USA; Quasi-experiment	Content: A multimodal counseling-based adolescent PA intervention Delivery mode: Combination of in-person counseling, digital reminders, and material incentives	1 Month	No	1.1; 2.2; 4.1; 5.1; 7.1; 10.1; 10.4	Accelerometry and global positioning system	Average daily minutes of MVPA	Very	ROBINS-I	M
Rubin et al., 2019 [15]	N = 66; Mean Age: IG = 10.0(1.0), CG = 9.2(1.0); BMI: 97th percentile; Country: USA; Quasi-experiment	Content: Parent-led PA intervention Delivery mode: Combination of in-person training, home-based activities, remote monitoring, and telephone support	24 W	SCT	1.1; 2.2; 2.3; 3.1; 4.1; 7.1; 10.1	Actigraph-GT3X	MVPA	Non	ROBINS-I	M
Ruotsalainen et al., 2015 [41]	N = 46; Age: 14.7(0.8); BMI: 28.1(5.7); Country: Finland; RCT	Content: Facebook-delivered lifestyle counseling and PA self-monitoring Delivery mode: Combination of home-based activities, digital support, and remote communication	12 W	No	1.1; 1.2; 1.4; 2.2; 2.3; 3.1; 4.1; 5.1; 7.1;	Polar Active PA monitor	MVPA	Non	The revised ROB 2	M
Suksong et al., 2024 [42]	N = 42; Mean Age: IG = 10.50(1.04), CG = 10.27(0.96); BMI: 27.18; Country: USA; RCT	Content: A walking intervention program based on self-efficacy theory Delivery mode: Combination of in-person instruction, self-monitoring, group-based activities, and remote support	8 W	SET	1.1; 2.2; 2.3; 3.1; 4.1; 6.1; 6.2; 8.1; 8.7; 10.1; 11.2	Actigraph-GT3X	Steps/MVPA	Very	The revised ROB 2	H
Wilson et al., 2012 [43]	N = 43; Age: 10 to 16; BMI: >95th percentile; Country: Canada; RCT	Content: Group-based exercise and self-regulatory intervention Delivery mode: Combination of in-person sessions, structured group activities, and ongoing medical and family support	12 W	SCT	1.1; 2.2; 2.3; 3.1; 4.1	The seven-day PA recall interview	MVPA	Quite	The revised ROB 2	H

Notes: BCTs: behavior change techniques; BMI: Body Mass Index; CG: control group; H: high risk; IG: intervention group; L: low risk; LPA: light physical activity; M: moderate; METs: metabolic equivalents; MVPA: moderate-to-vigorous physical activity; PA: physical activity; RCT: randomized controlled trial; ROB: risk of bias appraisal, a measure of study quality in assessing the potential for systematic errors; ROB 2: Cochrane Risk of Bias tool 2; ROBINS-I: Risk of Bias in Non-randomized Studies; SCT: self-cognitive theory; SDT: self-determinant theory; SET: self-efficacy theory; VPA: vigorous physical activity; W: week. 1.1 Goal setting (behavior); 1.2 Problem solving; 1.4 Action planning; 1.6 Discrepancy between current behavior and goal; 2.2 Feedback on behavior; 2.3 Self-monitoring of behavior; 3.1 Social support (unspecified); 3.3 Social support (emotional); 4.1 Instruction on how to perform the behavior; 5.1 Information about health consequences; 6.1 Demonstration of the behavior; 7.1 Prompts/cues; 8.1 Behavioral practice/rehearsal; 8.3 Habit formation; 8.7 Graded tasks; 9.1 Credible source; 9.2 Pros and cons; 10.1 Material incentive (behavior); 10.3 Non-specific reward; 10.4 Social reward; 10.5 Social incentive; 10.9 Self-reward; 11.2 Reduce negative emotions; 12.1 Restructuring the physical environment.

**Table 2:** BCTs applied by intervention effectiveness and promise ratio.

BCTs	Type of Intervention Efficiency				
	Very Potential (n = 6)	Quite Potential (n = 2)	Non Potential (n = 5)	Total (n = 13)	Promise Ratio ^†^ OR Number
1.1 Goal setting (behavior)	6	2	5	13	1.6
1.2 Problem solving	3	0	2	5	1.5
**1.4 Action planning** ^‡^	1	1	1	3	2
1.6 Discrepancy between current behavior and goal	0	1	0	1	1
2.2 Feedback on behavior	4	2	4	10	1.5
2.3 Self-monitoring of behavior	4	2	4	10	1.5
**3.1 Social support (unspecified)** ^‡^	3	1	2	6	2
3.3 Social support (emotional)	1	0	1	2	1
4.1 Instruction on how to perform behavior	5	2	4	11	1.75
5.1 Information about health consequences	4	0	3	7	1.33
6.1 Demonstration of the behavior	1	0	0	1	1
7.1 Prompts/cues	2	1	3	6	1
8.1 Behavioral practice/rehearsal	1	0	2	3	0.5
8.3 Habit formation	1	0	0	1	1
8.7 Graded tasks	1	0	0	1	1
9.1 Credible source	1	0	3	4	0.33
9.2 Pros and cons	1	0	0	1	1
**10.1 Material incentive (behavior)** ^‡^	3	0	1	4	3
10.3 Non-specific reward	0	1	0	1	1
10.4 Social reward	1	0	0	1	1
10.5 Social incentive	0	1	0	1	1
10.9 Self-reward	1	0	0	1	1
11.2 Reduce negative emotions	1	0	1	2	1
12.1 Restructuring the physical environment	1	0	0	1	1

Note: ^†^ The promise ratio refers to the ratio of times a behavior change technique (BCT) appears in very or quite promising interventions to the number of times it occurs in non-promising interventions. ^‡^ Rows in bold highlight BCTs with a promise ratio greater than two or those used in at least two promising interventions.

## Data Availability

The raw data from this study are available upon request from the corresponding author.

## Supplementary Materials

The following supporting information can be downloaded at: https://www.mdpi.com/article/10.3390/bs14121143/s1, S1: search strategy; S2: BCTs coding details; S3: ROB appraisal; S4: PRISMA checklist.
