# Supplementary material for: Behavior Change Techniques in Physical Activity Interventions Targeting Overweight and Obese Children and Adolescents: A Systematic Review
Source: Behav Sci (Basel). 2024 Nov 28;14(12):1143. doi: 10.3390/bs14121143 (PMC11673257; doi:10.3390/bs14121143)
Supplement: Supplementary file 1 [file behavsci-14-01143-s001.zip › S1 Searching strategy.pdf]

Searching Strategies

PubMed (5470)

((((("Adolescent"[Mesh]) OR ((((((((((Adolescent*[Title/Abstract]) OR (Teenager*[Title/Abstract])) OR (Youth*[Title/Abstract])) OR (Kids[Title/Abstract])) OR (Pupil*[Title/Abstract])) OR (Student*[Title/Abstract])) OR (Pre-adolescent*[Title/Abstract])) OR (Juvenile*[Title/Abstract])) OR (Tween*[Title/Abstract])) OR (Teen*[Title/Abstract]))) OR (("Child"[Mesh]) OR (((child[Title/Abstract]) OR (children[Title/Abstract])) OR (minor*[Title/Abstract])))) AND ((((obesity[Title/Abstract]) OR (obese[Title/Abstract])) OR (overweight[Title/Abstract])))) AND (("Exercise"[Mesh]) OR ((physical activit*[Title/Abstract]) AND (exercise*[Title/Abstract])))) AND ((((intervention*[Title/Abstract]) OR (program*[Title/Abstract])) OR (trial*[Title/Abstract])) OR (experiment*[Title/Abstract]))

Embase (8683)

#8. #3 AND #6 AND #7
#7. intervention: ab,ti OR program: ab,ti OR trial:ab,ti OR experiment:ab,ti
#6. #4 AND #5
#5. obesity:ab,ti OR obese:ab,ti OR overweight:ab,ti
#4. children:ab,ti OR adolescent:ab,ti OR teenager:ab,ti OR youth:ab,ti OR kid:ab,ti OR minor:ab,ti OR pupil:ab,ti OR student:ab,ti OR juvenile:ab,ti OR tween:ab,ti OR teen:ab,ti
#3. #1 OR #2
#2. exercise:ab,ti OR sport:ab,ti OR movement:ab,ti OR fitness:ab,ti OR activity:ab,ti OR recreation:ab,ti OR training:ab,ti OR workout:ab,ti OR 'physical education':ab,ti OR play:ab,ti OR dance:ab,ti
#1. 'physical activity'/exp OR 'physical activity'

Web of Science (10880)

#4. **Children** (Abstract) or **Adolescent** (Abstract) or **Teenager** (Abstract) or **Youth** (Abstract) or **Student** (Abstract) or **Juvenile** (Abstract) or **Tween** (Abstract) or **Teen** (Abstract) or **Pupil** (Abstract) or **Kid** (Abstract) or **Minor** (Abstract) and **Preprint Citation Index** (Exclude – Database)

#3.

Physical activity (Abstract) or **exercise** (Abstract) or **fitness** (Abstract) or **sport** (Abstract) or **movement** (Abstract) or **activity** (Abstract) or **recreation** (Abstract) or **training** (Abstract) or **workout** (Abstract) or **physical education**(Abstract) or **physical education** (Abstract) or **dance** (Abstract) and **Preprint Citation Index** (Exclude – Database)

#2.

obesity (Abstract) or **obese** (Abstract) or **overweight** (Abstract) and **Preprint Citation Index** (Exclude – Database)

#1.

intervention (Abstract) or **program** (Abstract) or **trial** (Abstract) or **experiment**(Abstract) and **Preprint Citation Index** (Exclude – Database)

#4 AND #3 AND #2 AND #1

Cochrane (6453)
#1:
(Children):ti,ab,kw OR (Adolescent):ti,ab,kw OR (Teenager):ti,ab,kw OR (Youth):ti,ab,kw OR (Kid):ti,ab,kw OR (Minor):ti,ab,kw OR (Pupil):ti,ab,kw OR (Student):ti,ab,kw OR (Juvenile):ti,ab,kw OR (Tween):ti,ab,kw OR (Teen):ti,ab,kw
#2:
(obesity):ti,ab,kw OR (obese):ti,ab,kw OR (overweight):ti,ab,kw
#3:
#1 AND #2
#4:
MeSH descriptor: [Exercise] explode all trees
#5:
(exercise):ti,ab,kw OR (physical activity):ti,ab,kw OR (fitness):ti,ab,kw OR (sport):ti,ab,kw OR (movement):ti,ab,kw OR (activity):ti,ab,kw OR (recreation):ti,ab,kw OR (training):ti,ab,kw OR (workout):ti,ab,kw OR (physical education):ti,ab,kw OR (play):ti,ab,kw OR (dance):ti,ab,kw
#6:
#4 OR #5
#7:
#3 AND #6

Psych info (2451)

(Abstract: Children OR Abstract: Adolescent OR Abstract: Teenager OR Abstract: Youth OR A bstract: Kid OR Abstract: Minor OR Abstract: Pupil OR Abstract: Student OR Abstract: Juvenile OR Abstract: Tween OR Abstract: Teen) AND (Abstract: obesity OR Abstract: obese OR Abstract: overweight) AND (Abstract: exercise OR Abstract: physical activity OR Abstract: fitness OR Abstract: sport OR Abstract: movement OR Abstract: activity OR Abstract: recreation OR Abstract: training OR Abstract: workout OR Abstract: physical education OR Abstract: play OR Abstract: dance) AND (Abstract: intervention OR Abstract: tria l OR Abstract: program OR Abstract: experiment)
