# Supplementary material for: Behavior Change Techniques in Physical Activity Interventions Targeting Overweight and Obese Children and Adolescents: A Systematic Review
Source: Behav Sci (Basel). 2024 Nov 28;14(12):1143. doi: 10.3390/bs14121143 (PMC11673257; doi:10.3390/bs14121143)
Supplement: Supplementary file 1 [file behavsci-14-01143-s001.zip › S2 ROB appraisal.pdf]

Table S1. Summary of ROB for RCT

Study	Design	Appraisal Tool	Randomisation process	Deviations from intended interventions	Missing outcome data	Bios in measurement of the outcome	Bias in selection of the reported result	Overall
Backlund et al., 2011a	RCT	The revised ROB 2	L	L	L	L	L	L
Backlund et al., 2011b	RCT	The revised ROB 2	C	L	H	L	L	H
Currie et al., 2017	Pilot RCT	The revised ROB 2	L	C	H	C	C	H
Gourlan et al., 2013	RCT	The revised ROB 2	L	L	L	L	L	L
Henderson et al., 2010	Pilot RCT	The revised ROB 2	L	C	L	C	C	C
Maloney et al., 2012	RCT	The revised ROB 2	C	C	C	C	C	C
Ruotsalainen et al., 2015	RCT	The revised ROB 2	L	C	C	C	C	C
Suksong et al., 2024	RCT	The revised ROB 2	L	C	H	C	C	H
Wilson et al., 2012	RCT	The revised ROB 2	L	C	H	C	H	H

Note : C: concerns; H: high risk; L: low risk; RCT: randomized controlled trial; ROB: risk of bias; ROB 2: The Cochrane Risk of Bias tool 2.

Table S2. Summary of ROB for non-RCT

Study	Design	Appraisal Tool	Bias due to confounding	Bias in selection of participants into the study	Bias in classification of interventions	Bias due to deviations from intended interventions	Bias due to missing data	Bias in measurement of outcomes	Bias in selection of the reported result	Overall
Cummings et al., 2022	Quasi-experimental	ROBINS-I	C	L	L	C	C	C	C	C
Morano et al., 2020	Quasi-experimental	ROBINS-I	C	L	L	C	C	C	C	C
Oreskovic et al., 2016	Quasi-experimental	ROBINS-I	C	L	L	C	L	C	C	C
Rubin et al., 2019	Quasi-experimental	ROBINS-I	C	L	L	C	C	C	C	C

Notes: C: concerns; H: high risk; L: low risk; ROBINS-I: the Risk Of Bias In Non-randomized Studies - of Interventions.
