# Supplementary material for: Behavior Change Techniques in Physical Activity Interventions Targeting Overweight and Obese Children and Adolescents: A Systematic Review
Source: Behav Sci (Basel). 2024 Nov 28;14(12):1143. doi: 10.3390/bs14121143 (PMC11673257; doi:10.3390/bs14121143)
Supplement: Supplementary file 1 [file behavsci-14-01143-s001.zip › S3 BCT coding details.pdf]

Table BCT coding details

Study	BCT Components	Evidence Description	Original Intervention Contents
Backlund et al., 2011a	1.1 Goal setting (behavior)	The intervention involves helping participants to set realistic and achievable goals related to lifestyle improvement.	"To help the children and parents to reflect on what realistic and reachable goals are, and how they can be used when improving lifestyle. Another goal was to help the participants understand the advantages of physical activity and healthy food habits. Children and parents practise to formulate goals that they perceive and reflect on what makes them realistic and reachable."
	2.2 Feedback on behavior	Participants receive feedback on their progress towards the 60 minutes/day physical activity goal.	" Information was given and discussion was held about why physical activity is important and how it can be a part of a healthy lifestyle."
	4.1 Instruction on how to perform the behavior	The intervention includes discussions and practical tasks on how to integrate physical activity, like using a pedometer, into daily life.	"To stimulate the children to achieve the gender adjusted step recommendation _ 12,000 steps/day for boys and _ 15,000 steps/day for girls. And to minimize the screen time to _ 2 h/day. Children and parents discuss in separate groups about how to integrate physical activity in the child's everyday life. The children also performed a practical task with pedometer."
	9.1 Credible source	The discussion about vigorous activities, including line dancing, is likely based on credible information sources, though this could be inferred depending on how the information was presented.	
	2.3 Self-monitoring of behavior	The use of an internet system for ongoing monitoring and logging of physical activity.	"The intervention was internet-based using the system PingPong. The second intervention year comprised four home assignments with different themes given to parents and children, some to be solved together and others to be solved by parents and children separately."
	8.1 Behavioral practice/rehearsal	Children are encouraged to practice and engage in physical activities recommended by other participants, reinforcing behavior through repetition.	

Study	BCT Components	Evidence Description	Original Intervention Contents
Backlund et al., 2011b	1.1 Goal setting (behavior)	The intervention involves helping participants to set realistic and achievable goals related to lifestyle improvement.	"To help the children and parents to reflect on what realistic and reachable goals are, and how they can be used when improving lifestyle. Another goal was to help the participants understand the advantages of physical activity and healthy food habits. Children and parents practise to formulate goals that they perceive and reflect on what makes them realistic and reachable."
	5.1 Information about health consequences	The intervention provides information on the importance of physical activity for maintaining a healthy lifestyle.	"To stimulate the children to achieve the physical activity goal, being active _ 60 min/day in at least moderate activity and in addition. Information was given and discussion was held about why physical activity is important and how it can be a part of a healthy lifestyle."
	2.3 Self-monitoring of behavior	Encourages self-monitoring of physical activity by counting steps, which helps participants gauge their daily activity levels.	"To take at least 12,000 or 15,000 steps a day."
	4.1 Instruction on how to perform the behavior	The intervention includes discussions and practical tasks on how to integrate physical activity, like using a pedometer, into daily life.	"To stimulate the children to achieve the gender adjusted step recommendation _ 12,000 steps/day for boys and _ 15,000 steps/day for girls. And to minimize the screen time to _ 2 h/day. Children and parents discuss in separate groups about how to integrate physical activity in the child's everyday life. The children also performed a practical task with pedometer."
	7.1 Prompts/cues	Pedometers are used as a tool to prompt and remind children to meet their daily step goals.	
	8.1 Behavioral practice/rehearsal	Encourages practicing the behaviors learned during the sessions, which reinforces the learning through actual application.	"Group sessions were designed mainly to suit the children, and a great deal of time was devoted to practical tasks with children and parents together."
	9.1 Credible source	Delivery of content by professionals adds credibility, encouraging adherence and trust in the prescribed activities and advice.	"Sessions led by a physical therapist and other health professionals."
Cummings et al., 2022	2.3 Self-monitoring of behavior	Participants were provided with Fitbit Charge HR devices to self-monitor their activity levels. They tracked their daily steps and active minutes using the Fitbit mobile application.	"Participants were provided a Fitbit Charge HR... to self-monitor their activity levels throughout the program."

Study	BCT Components	Evidence Description	Original Intervention Contents
	2.2 Feedback on behavior	SMS texts were sent to adolescents providing feedback on whether they met their activity goals the previous day, including praise or reminders.	“Tuesday through Friday, texts informed adolescents about whether they met their active minute or step goal the previous day...”
	1.1 Goal setting (behavior)	The study set clear daily and weekly activity goals, such as 60 minutes of moderate-to-vigorous activity or 10,000 steps per day.	“Adolescents were provided a daily goal of >60 active minutes or 10,000 steps of exercise per day...”
	7.1 Prompts/cues	Regular SMS messages acted as cues to remind and encourage participants to engage in physical activities.	"SMS text message content was standardized and focused specifically on activity goals."
	1.6 Discrepancy between current behavior and goal	Regular feedback compared the actual behavior (daily steps or active minutes) with the targeted goals, highlighting any discrepancies.	"Texts informed adolescents about whether they met their active minute or step goal the previous day."
	10.3 Non-specific reward (non-contingent)	Praise, a form of non-specific reward, was given for achieving daily goals, enhancing motivation.	"Texts...praised them if they met their goal."
	10.5 Reward (contingency management)	Monetary incentives were contingent on meeting set physical activity goals, serving as a motivator.	"Incentives for adherence to program weekly goals were loaded onto a reloadable debit card."
	10.1 Incentive (material)	Participants received monetary rewards for meeting activity goals, which were distributed via a reloadable debit card.	“Incentives for adherence to program weekly goals were loaded onto a reloadable debit card once per week...”
	4.1 Instruction on how to perform the behavior	Adolescents were given instructions on how to monitor their daily progress and achieve their activity goals.	“Adolescents were instructed on how to view daily progress toward exercise goals and incentives...”
Currie et al., 2017	1.1 Goal setting (behavior)	The researcher assisted adolescents in developing realistic goals related to increasing physical activity.	“...the researcher assisted the adolescents in developing realistic goals, an action plan, and strategies to overcome barriers.”
	1.4 Action planning	Adolescents were helped to develop an action plan and strategies to overcome barriers to physical activity.	“...developing realistic goals, an action plan, and strategies to overcome barriers.”

Study	BCT Components	Evidence Description	Original Intervention Contents
	2.2 Feedback on behavior	Telephone calls provided personalized feedback during discussions, encouraging change talk and resolving ambivalence.	"...telephone calls incorporated motivational interviewing techniques... to encourage change talk and resolve ambivalence."
	5.1 Information about health consequences	The intervention included discussion of the benefits of physical activity, which relates to the health consequences of increasing activity.	"...telephone discussion topics included... PA benefits."
	9.1 Credible source	Information was delivered by a credible source, here indicated by the specialized training that research staff underwent in motivational interviewing techniques.	"Research staff completed 7 h of training in motivational interviewing."
	11.2 Reduce prompts/cues	The discussions aimed at reducing barriers to physical activity, which involves identifying and minimizing environmental or personal cues that hinder behavior change.	"...reducing PA barriers..."
	3.3 Social support (emotional)	Telephone calls provided a form of emotional support, especially through reflective listening and encouragement.	"...telephone calls... incorporated motivational interviewing techniques... used open-ended questions and reflective listening to encourage the adolescents..."
	1.2 Problem solving	The intervention included discussing barriers to physical activity and strategies to overcome them, which is a form of problem solving.	"...strategies to overcome barriers."
Gourlan et al., 2013	1.1 Goal setting (behavior)	During Phase 3, the counselor and participant set realistic and achievable goals related to the alternative behaviors chosen by the adolescent.	"When alternative behavior had been chosen, the counsellor and participant set some goals that were realistic and achievable."
	1.2 Problem solving	In Phase 3, potential barriers to accomplishing the goals and strategies to overcome these barriers were discussed.	"Potential barriers to accomplishing the plan and strategies to overcome these barriers were also discussed."

Study	BCT Components	Evidence Description	Original Intervention Contents
	2.3 Self-monitoring of behavior	Participants were encouraged to monitor their behaviors (e.g., tracking weekly physical activity), which helps in self-regulation and increasing awareness of behavioral patterns.	"Providing self-monitoring of behaviour (e.g. self-monitoring habitual weekly PA) and providing feedback on behaviour."
	4.1 Instruction on how to perform the behavior	Throughout the sessions, especially in Phases 2 and 3, the counselor provided guidance on how to adopt alternative behaviors and overcome barriers.	"All options were first discussed... Then one or several alternative behaviors were selected depending on the participant's needs and aspirations."
	3.3 Social support (emotional)	Emotional support was given as the counselor engaged in reflective listening and affirmed the participant's autonomy, which helped to reduce resistance and resolve ambivalence.	"The counsellor's role was to elicit the participant's thoughts... Change talk was encouraged by exploring ambivalence and conflicting beliefs about behavior change."
	9.2 Pros and cons	During Phase 2, alternatives to current behaviors were discussed, weighing the pros and cons of these options to help the participant choose the best course of action.	"All options were first discussed... Then one or several alternative behaviors were selected depending on the participant's needs and aspirations."
	2.2 Feedback on behavior	Adolescents were given feedback regarding the discrepancies between their actual physical activity and the recommended levels, facilitating behavior awareness.	"...adolescents were given feedback about the differences between their actual behaviour and the recommendations."
	5.1 Information about health consequences	The benefits of physical activity were presented, focusing on physical health (e.g., weight loss, reduced health risks), psychological health (e.g., reduced stress and anxiety), and social benefits (e.g., making friends).	"...benefits of PA on physical health (e.g., weight loss, health risks reduction), psychological health (e.g., stress, anxiety), and social aspects (e.g., making friends) were presented."
Henderson et al., 2010	1.1 Goal setting (behavior)	The exercise consultation involved developing realistic goals and strategies to promote physical activity, customized to the participant's stage of change.	"...develop realistic goals and strategies to promote physical activity customized to the patient's stage of change."

Study	BCT Components	Evidence Description	Original Intervention Contents
	1.2 Problem solving	For participants in the contemplation stage, the consultation focused on strategies to move to the preparation stage, which involves identifying and solving barriers to increasing physical activity.	"For the patient in contemplation... the discussion focused on strategies to move to the preparation stage and incorporate small amounts of physical activity..."
	1.4 Action planning	This indicates the process of detailing what the participant plans to do to maintain or enhance their physical activity levels, including specifics of how they will execute these plans sustainably.	"For a patient in the action stage, on the other hand, the discussion focused on strategies to enhance his or her current physical activity levels in a sustainable manner."
	4.1 Instruction on how to perform the behavior	Participants received instructions on how to incorporate physical activity into their daily lives, particularly in ways that were sustainable and appropriate for their stage of change.	"...incorporate small amounts of physical activity into the participant's daily life... enhance... current physical activity levels in a sustainable manner."
	5.1 Information about health consequences	This involves discussing the health benefits and the physical and mental health consequences of physical activity or inactivity, tailored to the patient's readiness to change.	"The standardized exercise consultation consisted of a single one-to-one session designed to educate the participant, strengthen his/her motivation, and develop realistic goals and strategies to promote physical activity customized to the patient's stage of change."
	9.1 Credible source	The consultation was administered by a physician, which may have added credibility to the information provided about physical activity.	"...exercise consultation as applied by a physician..."
Maloney et al., 2012	2.2 Feedback on behavior	This mechanism involves providing participants with feedback on their behavior, specifically regarding their compliance with submitting activity logs.	"Contact was made to participants and their families if we did not receive their logs for over 2 weeks."
	12.1 Restructuring the physical environment	The provision of a Sony Playstation2 or, in some cases, a Nintendo Wii, along with DDR software, was intended to create a home environment conducive to engaging in physical activity (dancing).	"Participants who did not own a Sony PlaystationÒ2 received one at enrollment... we provided a NintendoÔ (Redmond, WA) WiiÔ version of the DDR game if that was the home console of their choice."

Study	BCT Components	Evidence Description	Original Intervention Contents
	7.1 Prompts/cues	Regular contact serves as a prompt or cue to remind participants to submit their activity logs, promoting compliance with study requirements.	"Contact was made to participants and their families if we did not receive their logs for over 2 weeks."
	3.1 Social support (unspecified)	The provision of two dance pads encouraged non-solo play, which may have involved social support from friends or family members participating in the activity together.	"Contact was made to participants and their families if we did not received their logs for over 2 weeks..."
	2.3 Self-monitoring of behavior	Participants were expected to maintain logs of their activity, which were monitored by the researchers.	Implied through the reference to receiving logs from participants.
	5.3 Information about social and environmental consequences	This aspect highlights how changes in the physical and social settings, like the provision of preferred gaming systems and settings that encourage group play, can influence participants' activity behaviors.	"DDR X with two dance pads to encourage non-solo play... providing a Nintendo Wii version of the DDR game if that was the home console of their choice."
Morano et al., 2020	1.1 Goal setting (behavior)	Children were engaged in goal-setting activities during weekly group meetings to improve their physical activity (PA).	"Behavioral skill training sessions included... goal-setting... to improve their PA."
	1.2 Problem solving	Problem-solving skills were taught during the behavioral management sessions to help children overcome barriers to physical activity.	"Behavioral skill training sessions included... problem-solving..."
	2.3 Self-monitoring of behavior	Children were encouraged to keep a weekly activity diary to track their physical activity outside of the organized sessions.	"Children were encouraged to be physically active outside of the organized sessions and invited to fill out an activity diary weekly."
	4.1 Instruction on how to perform the behavior	The exercise sessions and behavioral meetings provided instructions on how to engage in various physical activities and adopt healthy behaviors, including proper exercise techniques and healthy eating.	"The exercise program required... based on health-related fitness components... Behavioral skill training sessions included... facilitative self-talk, self-rewarding... children were encouraged to engage in healthy eating behaviors."

Study	BCT Components	Evidence Description	Original Intervention Contents
	5.1 Information about health consequences	The program emphasized health-related fitness components and provided education on healthy eating behaviors based on Italian nutritional guidelines, linking these behaviors to improved health outcomes.	"...based on health-related fitness components... Drawing on Italian nutritional guidelines and recommendations... children were encouraged to engage in healthy eating behaviors..."
	10.9 Self-reward	The intervention included training in self-reward strategies to reinforce positive physical activity behaviors.	"Behavioral skill training sessions included... self-rewarding..."
	3.1 Social support (unspecified)	Children were encouraged to recruit social support to help them maintain and increase their physical activity levels.	"Behavioral skill training sessions included... recruiting social support..."
	8.3 Habit formation	The program aimed to create a routine for children by requiring them to attend exercise sessions twice a week for seven months and encouraging regular physical activity outside of these sessions.	"The exercise program required twice-weekly attendance over 7 months... Children were encouraged to be physically active outside of the organized sessions..."
Oreskovic et al., 2016	1.1 Goal setting (behavior)	During the team meeting, the pediatrician and the participant agreed on a physical activity goal that involved using the surrounding built environment.	"The participant and pediatrician also decided on a physical activity goal, which the subject agreed to achieve two to three times per week and which involved a new use of the surrounding built environment."
	2.2 Feedback on behavior	Participants received feedback on their baseline average daily moderate-to-vigorous physical activity (MVPA) level during the team meeting.	"...they received feedback on their baseline average daily MVPA level."
	4.1 Instruction on how to perform the behavior	Participants received individualized counseling on how to increase their daily physical activity using their specific surrounding built environment, including practical advice on using parks, playgrounds, and other areas.	"...received individualized counseling based on their T1 data on how to increase their daily physical activity using their specific surrounding built environment..."

Study	BCT Components	Evidence Description	Original Intervention Contents
	5.1 Information about health consequences	Although not explicitly mentioned, counseling likely included information about the health benefits of increasing physical activity, a common component in such interventions.	Implied by the focus on increasing MVPA and using the built environment to achieve physical activity goals.
	7.1 Prompts/cues	Participants received weekly text messages and/or phone call reminders about their agreed-upon physical activity goal.	"Intervention participants received weekly text message and/or phone call reminders about their agreed on goal after their team meeting."
	10.1 Material incentive (behavior)	Participants received a physical activity promoting gift valued under $5 at T2, along with financial incentives ($5 for the subject and $10 for the family) for meeting their built environment goals.	"Adolescents in the intervention group also received a physical activity promoting gift valued under $5 at T2, along with financial incentives ($5 to the subject and $10 to the family) for meeting their agreed on built environment goal..."
	10.4 Social reward	Participants competed for a prize valued at several hundred dollars for achieving the greatest increase in MVPA over the course of the study, which served as a social reward.	"...competed for a prize valued at several hundred dollars for the participant achieving the greatest increase in MVPA over the course of the study."
Rubin et al., 2019	1.1 Goal setting (behavior)	The Active Play at Home curriculum included goal-oriented physical activities designed to engage participants in specific exercises four days per week over 24 weeks.	"The Active Play at Home curriculum included age-appropriate and goal-oriented physical activities that combined playground (twice a week) and active video games using the Nintendo Wii (twice a week)."
	2.3 Self-monitoring of behavior	Participants were provided with a checklist to complete at the end of each physical activity session, tracking the level of enjoyment, difficulty, and duration of the session.	"A checklist was included on the back of each PA session schedule, which was to be completed at the end of the session. Parents and children were asked to rate the level of enjoyment and difficulty of activities completed and to indicate the total duration of the session."
	2.2 Feedback on behavior	During a 12-week onsite visit, families received feedback on their progress to sustain motivation for participation.	"Families attended an onsite visit at 12 wk during which feedback was offered to families to sustain motivation for participation."

Study	BCT Components	Evidence Description	Original Intervention Contents
	3.1 Social support (unspecified)	The onsite visits and the supportive phone calls from the research team provide a form of social support aimed at encouraging ongoing participation and engagement in the physical activities outlined in the intervention.	"In addition, families attended an onsite visit at 12 wk during which feedback was offered to families to sustain motivation for participation."
	4.1 Instruction on how to perform the behavior	Participants were trained on the use of the curriculum during the baseline visit, which included instructions on how to engage in the physical activities and use the equipment.	"Participants were trained on the use of the curriculum during the baseline visit."
	7.1 Prompts/cues	Telephone calls from the research team served as prompts and cues to ensure participants stayed engaged with the program and to troubleshoot any issues with the curriculum.	"Parents received telephone calls from a member of the research team that followed a common script; PA counseling and troubleshooting related to curriculum implementation and motivation was provided during these telephone calls."
	10.1 Material incentive (behavior)	Youth participants received a $60 gift card if they completed 70% or more of the physical activity sessions over the 24 weeks.	"The youth received a $60 gift card to a department store if they completed Q70% of the PA sessions."
Ruotsalainen et al., 2015	4.1 Instruction on how to perform the behavior	The intervention provided informational support on physical activity and diet through themed posts on Facebook, offering guidance on how to maintain a physically active lifestyle.	"The informational support themes were published on Facebook © fan pages... (1) general information about physical activity and dietary recommendations... (6) their ability to maintain a physically active lifestyle."
	3.1 Social support (unspecified)	Social support was provided through the Facebook group, with a tutor (physiotherapist) and dietitian offering emotional and material support, as well as fostering peer interactions.	"Social support included emotional and material support and social support from the tutor, dietitian, parents, peers, and friends."
	1.2 Problem solving	The intervention included behavioral management skills such as problem-solving related to barriers to physical activity, shared via posts in the Facebook groups.	"Behavioural management skills included problem solving related to the barriers to being physically active; motivating questions and ideas were shared via posts."

Study	BCT Components	Evidence Description	Original Intervention Contents
	1.4 Action planning	The opportunity for adolescents and their parents to create personalized exercise programs based on their needs fits the BCT 1.4 Action planning, where participants plan when, where, and how they will perform the behavior.	"Adolescents and their parents also had the opportunity to make tailored exercise programme suggestions."
	2.3 Self-monitoring of behavior	Adolescents in the Facebook plus Activity Monitor group (Fb + Act) were given Polar Active physical activity monitors to self-monitor their daily physical activity and sedentary time.	"The Fb + Act group used the monitor on their wrists for 12 weeks. The monitor continuously measured their daily activity at different activity levels..."
	2.2 Feedback on behavior	The activity monitor provided real-time feedback on daily physical activity levels, including intensity, duration, and sedentary time, showing progress in relation to recommended levels.	"The monitor showed the user's individual daily steps and total daily energy consumption in calories (Kcal)... a bar showing the amount of activity in relation to a recommended level."
	5.1 Information about health consequences	The intervention provided information about the health consequences of physical activity and diet, aiming to reduce BMI and promote overall health.	"The informational support themes were published on Facebook... (1) general information about physical activity and dietary recommendations."
	7.1 Prompts/cues	The tutor consistently provided cues and reminders via Facebook posts, which helped participants stay engaged in their physical activity routines. This fits 8.7	"The tutor shared material and read posts, commented on them, and answered questions."
	1.1 Goal setting (behavior)	The intervention likely involved setting goals for physical activity, as participants were guided by tailored exercise program suggestions and self-monitoring tools.	Implied by the tailored exercise program and use of self-monitoring devices.
Suksong et al., 2024	1.1 Goal setting (behavior)	Children were asked to set a walking goal of 6,000 steps in weeks 1 and 2, with an incremental increase of 1,000 steps every two weeks.	"The students were asked to set the goal of walking 6000 steps in weeks 1 and 2 and increase it by 1000 steps every two weeks."

Study	BCT Components	Evidence Description	Original Intervention Contents
	2.3 Self-monitoring of behavior	Children were given a pedometer to track their daily steps and were asked to record these in a step diary, which they reported to the researcher.	"Each member of the intervention group received an SW-701 YAMAX DIGI-WALKER pedometer... recorded their daily steps after school time in a step diary... and reported the results to the researcher the next day."
	2.2 Feedback on behavior	Children received positive feedback on their steps each week, which was aimed at reinforcing their progress and motivating continued effort.	"They receive positive feedback on their steps each week."
	4.1 Instruction on how to perform the behavior	Children were provided with detailed instructions on how to use the pedometer and record their steps accurately in the diary.	"Each member of the intervention group received an SW-701 YAMAX DIGI-WALKER pedometer... along with an explanation of how to use the pedometer and record the data."
	6.1 Demonstration of the behavior	Live modeling was used by the researcher to demonstrate the methods and benefits of physical activity to enhance knowledge and motivation.	"The researcher created motivation to change the health behavior of the intervention group by using verbal persuasion and live modeling..."
	8.1 Behavioral practice/rehearsal	Children tried out the pedometer for one week before fully participating in the program, allowing them to practice the behavior of tracking steps.	"The children received the pedometer and tried it out for one week before participating in the program."
	8.7 Graded tasks	The program involved gradually increasing the step goal by 1,000 steps every two weeks, which allowed for a progressive increase in physical activity.	"...increase it by 1000 steps every two weeks."
	6.2 Social comparison	Children were informed about the steps taken by role models, and group competitions were held to encourage comparisons and motivate increased activity levels.	"The practical strategies for facilitating vicarious experiences in this intervention were as follows: 1) children received information about the steps of role models... There was a competition for the combined number of steps taken by each group each week during weeks 5-8."
	3.1 Social support (unspecified)	Children were placed in groups to create mutual incentives, motivation, and support for achieving their step goals.	"Children were divided into groups of 3-4 people, and goals were set for the group to create mutual incentives within the group and to create motivation."
	10.1 Material incentive (behavior)	Groups competed for the highest number of steps, with the winning group receiving a	"The group with the highest number of steps was identified, and they won and received a prize."

Study	BCT Components	Evidence Description	Original Intervention Contents
		prize, which served as a social reward to motivate participation.	
	11.2 Reduce negative emotions	Stress and negative emotions were reduced by advising children to start with minimal steps and gradually increase their activity level, thereby reducing the pressure to perform.	"Stress and negative emotions were reduced by advising children to take steps at minimum levels and increasing the number of steps taken at a low rate each week."
Wilson et al., 2012	1.1 Goal setting (behavior)	Participants were randomized to engage in either moderate or vigorous intensity exercise, which likely involved setting specific exercise goals as part of the structured program.	"All participants were randomized to take either metformin or placebo, and then randomized to engage in either moderate or vigorous intensity exercise (aerobic and resistance exercises)."
	4.1 Instruction on how to perform the behavior	Participants received instruction on how to engage in aerobic and resistance exercises during the structured exercise sessions at a local community center.	"...engaged in weekly exercise sessions at a local community centre..."
	2.2 Feedback on behavior	Feedback was likely provided during the weekly GMCB (Group-based Cognitive Behavioral) sessions, which were designed to support the exercise intervention.	"Everyone participated in identical weekly GMCB sessions for the first 12 weeks of the two-year intervention."
	3.1 Social support (unspecified)	Social support was provided through weekly GMCB sessions, family sessions with a dietician and social worker, and group exercise sessions, creating a supportive environment for behavior change.	"...engaged in weekly exercise sessions at a local community centre, attended family sessions with a dietician and a social worker..."
	2.3 Self-monitoring of behavior	The study included follow-up assessments of independent physical activity, suggesting that participants were encouraged to monitor their own activity levels outside of the structured sessions.	"The current study focuses on the feasibility of the 12-week exercise plus GMCB intervention and includes post-intervention follow-up assessments of independent physical activity..."
